# Prevalence of Selective Serotonin Reuptake Inhibitor Use Among Pregnant Women From 2017 to 2020 in King Abdulaziz Medical City, Jeddah, Saudi Arabia: A Retrospective Study

**DOI:** 10.7759/cureus.47745

**Published:** 2023-10-26

**Authors:** Alqassem Y Hakami, Rami Ghazi Ahmad, Mustafa M Bukhari, Mohammed Assaf Almalki, Mamdoh M Ahmed, Mohammed M Alghamdi, Mulham A Kalantan, Khalil M Alsulami

**Affiliations:** 1 College of Medicine, King Saud Bin Abdulaziz University for Health Sciences, Jeddah, SAU; 2 Research Office, King Abdullah International Medical Research Center, Jeddah, SAU; 3 Psychiatry Section, Medicine Department, Ministry of National Guard - Health Affairs, Jeddah, SAU

**Keywords:** selective serotonin reuptake inhibitor (ssri), depression, antidepressant, pregnant, serotonin

## Abstract

Background: Perinatal depression is a mental health disorder that is associated with feelings of hopelessness, despair, and lack of motivation. Its effects on pregnant women are not limited to hemorrhage and hypertension and may lead to maternal mortality. As a result, maternal antidepressant usage during pregnancy has rapidly increased in the United States. Selective serotonin reuptake inhibitors (SSRIs) are considered one of the most prescribed antidepressants. Thus, this study aims to measure the prevalence of SSRI use during pregnancy.

Methods: A retrospective cross-sectional study was carried out in King Abdulaziz Medical City, Jeddah (KAMC-J), Saudi Arabia. The population consisted of all pregnant women aged 18 or older from the period of January 2017 to December 2020 (N=13484). The sampling technique was non-probability consecutive sampling.

Results: The study included 13,484 pregnant women, and further analysis revealed that 62 (0.459%) were exposed to at least one type of antidepressant during pregnancy. Of these, 12 (19.35%) had used more than one class of antidepressants. The majority of the sample, comprising 39 (62.90%) women, were between 34 and 44 years old. Furthermore, SSRIs were found to be the most commonly used antidepressant (41, 66.13%). In addition, fluoxetine was the most frequently prescribed antidepressant, with 23 (37.10%) patients receiving this medication. The dosage did not exceed 20 mg for the majority of the patients on SSRIs.

Conclusion: This study measured the prevalence and patterns of SSRIs and use of different antidepressant classes during pregnancy. After calculating the prevalence of each class of antidepressants among 62 pregnant women exposed to antidepressants, the analysis concluded that SSRIs are the most prescribed antidepressant during pregnancy. This study contributes to the growing body of literature on the use of antidepressants during pregnancy and highlights the need for ongoing research in this area.

## Introduction

The global prevalence of prenatal anxiety and depression disorders in women is estimated to be 20% and 15%, respectively [1,2]. Studies have produced varying estimates of the prevalence of prenatal depression and anxiety based on countries’ income. The incidence of depression during pregnancy is estimated to be between 7% and 20% in high-income countries [3-8]. Meanwhile, rates of 20% or more have been documented in low- and middle-income countries, although less research has been conducted in these areas [9-12]. Furthermore, several studies have investigated the prevalence of anxiety and depression with respect to the specific trimesters during which they manifest. A meta-analysis by Dennis et al. showed that prenatal anxiety was more common in the third trimester followed by the second trimester and least common in the first trimester [2]. In addition, a systematic review by Okagbue et al. revealed that prenatal depression was more frequent in the third trimester and least frequent in the second trimester [13].

Prenatal depression, defined as depression that occurs during pregnancy, is a contributing factor to postpartum depression, which has been shown to have severe consequences on infants' and mothers' mental health [14]. Unrecognized and/or untreated prenatal depression may have an impact on both infants and mothers. Prenatal depression was shown to elevate the risk of premature birth, surgical delivery, and low birth weight [15,16]. Low birth weight and premature delivery are the major causes of infant deaths [17]. A study reported the association between maternal depression and significant pregnancy complications, such as reduced uterine artery blood flow, pre-eclampsia, pregnancy-induced hypertension, and antepartum hemorrhage [18]. As a result, screening for depression and its causes among pregnant women is highly recommended. Moreover, prevalence studies reported that selective serotonin reuptake inhibitors (SSRIs) are considered the most common antidepressants used during pregnancy [19]. Serotonin influences some of the activities in the adult brain, such as learning, memory, mood management, anxiety, fear, social, reproductive behavior, and higher mental functions [20]. Therefore, inhibiting the reuptake of this monoamine can alleviate the manifestations of mild to severe depression [21].

The management of depression in pregnancy is challenging and complicated. A "black box" warning was issued by the U.S. Food and Drug Administration (FDA) in 2004 regarding the use of SSRIs. The warning was issued due to the increased risk of suicidal ideation and behaviors in adolescents treated with SSRIs for major depressive disorder (MDD) [22]. Some studies reported an association between SSRIs and persistent pulmonary hypertension (PPHN) [23,24]. However, a study found no relation between SSRI use in pregnancy and PPHN [25]. Moreover, women who received SSRIs late in pregnancy had a higher chance of delivering small-for-gestational-age infants (SGA infants) [26]. In addition, several studies have shown an association between first-trimester exposure to SSRIs and some congenital disabilities [27-29]. The risk of preterm birth (PTB), identified as delivery before 37 weeks of gestation, is elevated with SSRI use and depression [30]. Several studies reported a higher prevalence of preterm birth in depressed pregnant women treated with SSRIs than in a control group [30-32].

Several studies that evaluated the prevalence of SSRI use among pregnant women showed similar outcomes. A study from New Zealand reported a decrease in antidepressant use in pregnant women from 2.7% to 2.6% following the first trimester [33]. Similarly, a previous cohort study found a reduction in antidepressant use over pregnancy from 23.9 per 1000 in the first trimester to 10.4 and 8.4 per 1000 pregnancies in the second and third trimesters, respectively [34]. Moreover, research published in Denmark investigated the prevalence of antidepressant exposure among pregnant women between 1997 and 2010. This latter study revealed an increase in exposure from 0.2% to 3.2% and a reduction in antidepressant exposure associated with pregnancy recognition [35]. In addition, a project in Nordic countries that explored the utilization of SSRIs among pregnant women reported a percentage of 3.3% out of 1,162,470 pregnant women who had been exposed to SSRIs. Moreover, in this study, due to concerns regarding the possible risk to the fetus, a high percentage of pregnant women quit using antidepressant medications upon the recognition of pregnancy [36]. Furthermore, a study in the United States discovered that 6% of 1,895,519 pregnant women were exposed to SSRIs. In addition, SSRI usage decreased in the later stages of pregnancy compared to the first term of pregnancy and before pregnancy [37].

Despite these studies, up to the authors' knowledge, there is a lack of information regarding the prevalence of antidepressant consumption among pregnant women in Saudi Arabia. Therefore, this study aims to address this research gap by identifying patterns and measuring the prevalence of SSRI use during pregnancy in a cross-sectional retrospective manner, including all pregnancies between January 2017 and December 2020 in King Abdulaziz Medical City in Jeddah (KAMC-J), Saudi Arabia.

## Materials and methods

Design and setting

A retrospective cross-sectional study was carried out in KAMC-J, Saudi Arabia. The study population consisted of all pregnant women at KAMC-J from January 2017 to December 2020 (N=13484). The study used a non-probability consecutive sampling technique. To ensure data accuracy, a list of all pregnant women who visited the psychiatry department at KAMC-J was provided and filtered by the medical record department to include pregnant women with psychiatric disorders and depressive disorders and those over 18 years old. Following that, the authors utilized the filtered list to select participants who met the inclusion criteria, which required participants to be pregnant women aged 18 or older who had been exposed to at least one antidepressant during pregnancy. The data was extracted from the BESTCare system (patient data system) by the authors, who recorded variables, such as age, diagnosis of depression, type of antidepressant medication, dosage, and frequency of use during pregnancy on the data collection sheet. Pregnant women under the age of 18, pregnant women who had not been exposed to an antidepressant, and pregnant women without depressive and psychiatric disorders were excluded from the study. The required sample size was calculated using the Raosoft website with a 95% confidence interval (CI), a 50% response distribution, and a 5% margin of error. The minimal sample size required was calculated to be 377.

Statistical analysis

The study used IBM SPSS Statistics for Windows (released 2020; IBM Corp., Armonk, New York, United States) for data analysis. The number of pregnant women who filled at least one prescription or were actively using an antidepressant during pregnancy in the study group was used to calculate the prevalence of antidepressant usage during pregnancy. Patients were divided according to their age into the following groups: 24 and below, 25-34, 35-44, and 45 and above. Furthermore, antidepressants were divided into groups based on single or multiple drugs used by the patients. To describe categorical data, frequency and percentages were used, which were the drug groups, age, dosage, frequency, and each drug individually (i.e., SSRIs, serotonin and norepinephrine reuptake inhibitors (SNRIs), atypical antidepressants, and tricyclic antidepressants (TCAs)). Moreover, Fisher's exact test was used to compare categorical data, and p-value of <0.05 was considered statistically significant.

Ethical consideration

The patients’ data were presented using serial code numbers, and no personal information or any patient identifiers were reported. The researchers were the only ones with access to the collected data, and the Principal Investigator (PI) ensured the privacy and confidentiality of the subjects. All data, both electronic and hard copies, were stored within the premises of National Guard Health Affairs (NGHA) and accessible only by the PI. Approval was granted by the King Abdullah International Medical Research Center (KAIMRC) Institutional Review Board (study number: SP21J-107-03).

## Results

The study included 13,484 women, and only 62 (0.459%) were exposed to at least one type of antidepressant during pregnancy; 19.35% used more than one class of antidepressants. Upon dividing patients according to age, 6.45% were at the age of 24 years and below, 25.81% were between the ages of 25 and 34 years, 62.90% were between the ages of 35 and 44 years, and 4.84% were at the age of 45 years and above (Table 1). However, there was no variation between age groups and the consumption of antidepressants. Furthermore, Fisher's exact test revealed no significant association between all age groups and different types of antidepressants, with a p-value >0.05.

**Table 1:** Prevalence of antidepressant use among age groups.

Age	N	N(%)	P-value
35-44	39	62.90%	P-value > 0.05
25-34	16	25.81%	
24 and below	4	6.45%	
45 and above	3	4.84%	

Drug groups

**Figure 1:**
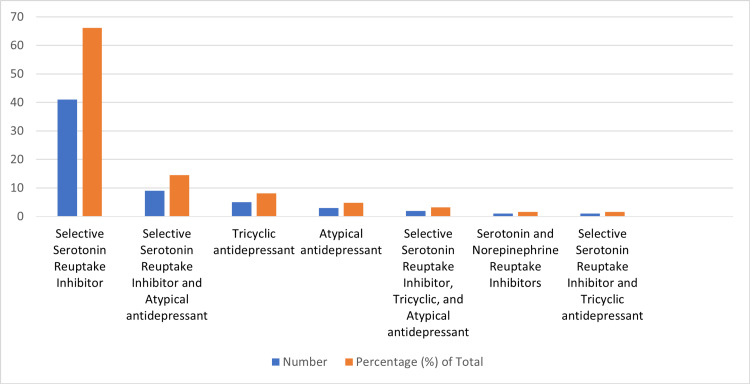
Prevalence of antidepressant drug usage during pregnancy. The majority of pregnant women on antidepressants used SSRIs, followed by TCAs and atypical antidepressants.

Among the 62 pregnant women included in the study, 66.13% used SSRIs, such as fluoxetine and citalopram, while 8.06% used TCAs (amitriptyline), 4.84% used atypical antidepressants (mirtazapine), and 1.61% used SNRIs (venlafaxine). A proportion (19.36%) of the pregnant women were on multiple classes of antidepressants, 14.52% of which used a combination of SSRIs and atypical antidepressants; 3.23% of which used a combination of SSRIs, TCAs, and atypical antidepressants; and 1.61% of which used a combination of SSRIs and TCAs (Figure 1). In total, 77.42% of the pregnant women used only one medication. Moreover, 37.10% used fluoxetine, 20.97% used citalopram, 8.06% used amitriptyline, 4.84% used mirtazapine, 3.23% used escitalopram, 1.61% used fluvoxamine, and 1.61% used venlafaxine (Figure 1, Table 2). Among the patients who used different classes of antidepressants, 8.06% combined fluoxetine and mirtazapine; 6.45% combined citalopram and mirtazapine; 3.23% combined citalopram, amitriptyline, and mirtazapine; and 1.61% combined fluoxetine and amitriptyline. Some patients (3.23%) combined two types of SSRIs, with 1.61% using fluoxetine and citalopram and the other 1.61% using fluoxetine and paroxetine (Table 2).

**Table 2:** Prevalence of antidepressants.

Drug	N	N(%)
Fluoxetine	23	37.10%
Citalopram	13	20.97%
Amitriptyline	5	8.06%
Fluoxetine and mirtazapine	5	8.06%
Citalopram and mirtazapine	4	6.45%
Mirtazapine	3	4.84%
Citalopram, amitriptyline, and mirtazapine	2	3.23%
Escitalopram	2	3.23%
Fluoxetine and amitriptyline	1	1.61%
Fluoxetine and citalopram	1	1.61%
Fluoxetine and paroxetine	1	1.61%
Fluvoxamine	1	1.61%
Venlafaxine	1	1.61%

Frequency and dosage of antidepressants

Regarding the dosage of antidepressant medications, 77.42% of the patients who used fluoxetine were on 20 mg dosage, 12.90% patients were on 40 mg, 6.45% were on 60 mg, and 3.23% patient’s dosage was missing. A large percentage (87.10%) of the patients used fluoxetine daily, and 12.90% patients’ frequencies were missing. Patients using citalopram (65%) were on 20 mg, 25% were on 10 mg, 5% was on 20 mg, and 5% was on 40 mg. Moreover, 85% of the patients used citalopram daily, while 15% patients' frequencies were missing. A proportion (78.57%) of the patients using mirtazapine used 15 mg, 7.14% was on 7.5 mg, 7.14% was on 30 mg, and 7.14% patient's dosage was missing. Meanwhile, 57.14% patients used mirtazapine daily, and 42.86% patients' frequencies were missing. A percentage (37.50%) of the patients who used amitriptyline were on 10 mg, 37.50% were on 50 mg, 12.50% was on 75 mg, and 12.50% were on 25 mg. All patients who used amitriptyline used it daily. One-hundred percent of escitalopram users were on 10 mg daily. The only patient using fluvoxamine was on 50 mg daily. The only patient using paroxetine was on 20 mg daily. The only patient using venlafaxine was on 150 mg daily (Table 3 and Table 4).

**Table 3:** Frequency of drug usage.

Drug	Daily	Missing
Fluoxetine	27 (87.10%)	4 (12.90%)
Citalopram	17 (85%)	3 (15%)
Mirtazapine	8 (57.14%)	6 (42.86%)
Amitriptyline	8 (100%)	0
Escitalopram	2 (100%)	0
Fluvoxamine	1 (100%)	0
Paroxetine	1 (100%)	0
Venlafaxine	1 (100%)	0

**Table 4:** Dosage of antidepressants during pregnancy.

Drug	Dose	N	N(%)
Fluoxetine	20 mg	24	77.42%
	40 mg	4	12.90%
	60 mg	2	6.45%
	Missing (dose)	1	3.23%
Citalopram	20 mg	13	65%
	10 mg	5	25%
	30 mg	1	5%
	40 mg	1	5%
Mirtazapine	15 mg	11	78.57%
	30 mg	1	7.14%
	7.5 mg	1	7.14%
	Missing (dose)	1	7.14%
Amitriptyline	10 mg	3	37.50%
	50 mg	3	37.50%
	75 mg	1	12.50%
	25 mg	1	12.50%
Escitalopram	10 mg	2	100%
Fluvoxamine	50 mg	1	100%
Paroxetine	20 mg	1	100%
Venlafaxine	150 mg	1	100%

## Discussion

A study conducted in the United States demonstrated that the effects of depression on pregnant women may exceed the impact of hemorrhage and hypertension and could lead to maternal mortality [38]. Moreover, the prevalence of maternal antidepressant usage during pregnancy have grown globally over the last 10 years, and the most prescribed antidepressants are SSRIs [39]. To the best of our knowledge, there is a lack of studies regarding the prevalence of antidepressant use among pregnant women in Saudi Arabia. This research aimed to calculate the prevalence of SSRI exposure during pregnancy in a cross-sectional retrospective manner, including pregnant women between January 2017 and December 2020 in KAMC-J, Saudi Arabia. This study indicates that the most prevalent class of antidepressants during pregnancy is SSRIs.

The statistical analysis confirms that among 62 women who used antidepressants during their pregnancy, 66.13% used at least one SSRI drug. The two most used medications were fluoxetine and citalopram, prescribed for around 50% and 32.3% of the sample, respectively. The observed results in this study regarding the SSRI usage during pregnancy were in line to the results in a study conducted by Zoega et al. in which SSRIs, citalopram, fluoxetine, and escitalopram were among the most commonly used antidepressants during pregnancy [36]. Another study done in the United States by Andrade et al. in 2016 reported that citalopram and fluoxetine as one of the most utilized antidepressants in pregnancy [37]. Moreover, data in this research found that there was no association between age groups and the use of antidepressants during pregnancy. A report by Zoega et al. found that antidepressants were mainly used in younger pregnant women aged 34 and less [36]. Conversely, some studies found that older women were more likely to use an antidepressant during pregnancy [40-42]. The similarity of results of this research with previous research might be due to the similarity of guidelines used in treating depressed pregnant patients. For example, the report on the management of depression during pregnancy by Yonkers et al. stated that SSRIs are the most prescribed class by physicians, while TCAs were among the least prescribed [43].

Furthermore, the safety of antidepressants played an important role in determining the percentages in data. The results in this research demonstrated that fluoxetine was the most used antidepressant. Several studies showed that fluoxetine use early in pregnancy has no significant association with major congenital malformations [44,45]. Another study from Australia showed that the use of fluoxetine early in pregnancy had higher chances of gastrointestinal tract malformations and malformations of the neck, ear, and face [46]. Moreover, fluoxetine showed higher chances of omphalocele, anencephaly, and craniosynostosis in infants compared to the control group [27]. Several cohort studies revealed that fluoxetine use early in pregnancy was associated with cardiac malformations [47-49]. Citalopram was the second most used antidepressant according to this research. Maternal use of citalopram in the early phases of pregnancy was associated with anomalies of the urinary system [46,48,50], gastrointestinal system [48,50], neural tube defects [51], omphalocele, anencephaly, and craniosynostosis [27]. In addition, citalopram usage in the first phases of pregnancy showed a higher risk of congenital heart defects [48,52,53].

By contrast, multiple studies demonstrated that in-utero exposure to SSRIs had no increased chance of major or specific cardiac malformations [27,28,51,54]. However, paroxetine was linked to a higher chance of cardiac malformations [54-56]. In addition, several studies attempted to study the association of fluoxetine and citalopram with miscarriages, and there was insignificant evidence of the association [44,57-63]. However, one study demonstrated that the underlying maternal depression played a significant role in miscarriage as the pregnant women had discontinued the use of antidepressants three to 12 months before the pregnancy [60]. Furthermore, this research showed that the majority of medications were prescribed at low doses; this could be attributed to antidepressant use guidelines, as physicians should start from the lowest dose possible to avoid side effects [64]. For example, it was reported that a high dose of venlafaxine (70 mg/kg) caused mild maternal intoxication [65,66]. Regardless of the risk-benefit assessment made at the start of the pregnancy, the best treatment options for pregnant women with depressive symptoms should be extensively reassessed.

The hospital's database system can provide accurate details on the types, doses, and names of antidepressants used by pregnant women; this results in obtaining accurate information under ethical standards that are not affected by recall bias. However, the study has some potential limitations. Compared to other studies, the sample size appears to be smaller because it is limited to women who gave birth in King Abdulaziz Medical City in Jeddah. Some of the pregnant mothers might be from rural areas, which could result in their deliveries and follow-ups occurring in other hospitals. Moreover, data regarding the complications of antidepressants on mothers and infants were unavailable due to discontinuation of follow-ups. Furthermore, some of the dosages and frequencies were missing from the database. Despite these limitations, the results of this study can serve as preliminary data for identifying the prevalence of antidepressant use among pregnant women. In addition, it highlights the need for psychiatrists and obstetricians-gynecologists to collaborate in the screening for depression in pregnant women and ensuring that they receive appropriate care and monitoring to minimize risks to both mothers and fetuses. The study also emphasizes the importance of educating pregnant women about the potential risks and benefits of antidepressants use during pregnancy and encourage them to discuss their medication use with their healthcare providers.

## Conclusions

This study aimed to identify patterns and measure the prevalence of SSRIs and different antidepressant classes during pregnancy. The statistical analysis confirms that among 62 women who used antidepressants during their pregnancy, SSRIs are the most used drugs. The two most prescribed medications are fluoxetine and citalopram. Moreover, future studies are warranted to measure the prevalence of SSRI use among pregnant women over a 10-year period to obtain a larger sample size. Nevertheless, these results indicate the need for future research focusing on the side effects of SSRIs and other antidepressants on pregnant women due to the sensitivity of the population and the lack of local studies on the prevalence and complications.
